# Angiogenesis-related protein expression in bevacizumab-treated metastatic colorectal cancer: NOTCH1 detrimental to overall survival

**DOI:** 10.1186/s12885-015-1648-4

**Published:** 2015-09-22

**Authors:** Tadeu Ferreira Paiva, Victor Hugo Fonseca de Jesus, Raul Amorim Marques, Alexandre André Balieiro Anastácio da Costa, Mariana Petaccia de Macedo, Patricia Maria Peresi, Aline Damascena, Benedito Mauro Rossi, Maria Dirlei Begnami, Vladmir Cláudio Cordeiro de Lima

**Affiliations:** 1 Department of Medical Oncology, A. C. Camargo Cancer Center, São Paulo, Brazil; 2 Department of Pathology, A. C. Camargo Cancer Center, São Paulo, Brazil; 3 Department of Statistics, Centro Internacional de Pesquisa e Ensino - Fundação Antônio Prudente, São Paulo, Brazil; 4 PhD/MSc Program, Hospital Sírio-Libanês, São Paulo, Brazil; 5 Department of Clinical Oncology, 1° Subsolo, Edifício Hilda Jacob R. Prof. Antônio Prudente, 211, São Paulo, ZC 01509-900 Brazil

**Keywords:** Colorectal cancer, Angiogenesis, NOTCH1, Survival

## Abstract

**Background:**

The development of targeted therapies has undoubtedly broadened therapeutic options for patients with colorectal cancer (CRC). The use of bevacizumab to reduce angiogenesis has been associated with improved clinical outcomes. However, an urgent need for prognostic/predictive biomarkers for anti-angiogenic therapies still exists.

**Methods:**

Clinical data of 105 CRC patients treated with bevacizumab in conjunction with chemotherapy were analyzed. The expression of vascular endothelial growth factor (VEGF) receptors, NOTCH1 receptor and its ligand DLL4 were determined by immunohistochemistry. Tumor samples were arranged on a tissue microarray. The association between protein expression and clinicopathological characteristics and outcomes was determined.

**Results:**

Bevacizumab was administered as a first-line of treatment in 70.5 % of our cases. The median progression-free survival (PFS) was 10.2 months. The median overall survival (OS) of the total cohort was 24.4 months. Bevacizumab, as the first-line of treatment, and the presence of liver metastasis were independently associated with objective response rate. Membrane VEGFR1 and VEGFR3 expressions were associated with the presence of lung metastasis; interestingly, VEGFR3 was associated with less liver metastasis. NOTCH1 expression was associated with lymph node metastasis. There was a trend toward association between improved PFS and lower NOTCH1 expression (*p* = 0.06). Improved OS was significantly associated with lower NOTCH1 expression (*p* = 0.01). In a multivariate analysis, ECOG (Eastern Cooperative Oncology Group) performance status, liver metastasis, histological grade, and NOTCH1 expression were independently associated with OS.

**Conclusion:**

Our findings illustrated the expression profile of angiogenesis-related proteins and their association with clinicopathological characteristics and outcomes. NOTCH1 expression is a detrimental prognostic factor in metastatic CRC patients treated with chemotherapy plus bevacizumab.

**Electronic supplementary material:**

The online version of this article (doi:10.1186/s12885-015-1648-4) contains supplementary material, which is available to authorized users.

## Background

Colorectal cancer (CRC) is the fourth most common cause of death worldwide, accounting for 694,000 deaths in 2012. CRC incidence is higher in men than in women, being the third most common cancer in men and the second in women [1]. Almost 50 % of patients will develop metastases and ~25 % already have metastasis at diagnosis [2]. Although CRC incidence and mortality rates vary markedly around the world, CRC is mainly a disease of developed Western countries. For 2015, it has been estimated that the United States will have 132,700 new cases of CRC and 49,700 related deaths [3]. In Brazil, 32,600 new cases of colorectal cancer were expected in 2014 (http://www.inca.gov.br/estimativa/2014/).

Angiogenesis induction is pivotal to tumor growth and metastases. From a molecular perspective, angiogenesis is triggered by hypoxia, cytokines, oncogenes activating mutations, growth factors and hormones that directly or indirectly promote the production and release of an array of proteins genetically and functionally related to VEGFA (vascular endothelial growth factor A). Subsequently, these proteins bind to and activate specific membrane tyrosine-kinase receptors (VRGFR1, VEGFR2 and VEGFR3) [4].

Similarly, a family of membrane bound receptors related to the protein NOTCH (NOTCH1, NOTCH2, NOTCH3 and NOTCH4) interact with membrane bound ligands (JAGGED-1 or JAG1, JAG2, delta-like-1 or DLL1, DLL3 and DLL4) and act to regulate cell proliferation, differentiation and apoptosis, as well as angiogenesis and tumor cell migration [5, 6]. Some data suggest that VEGFR2 activation by VEGFA upregulates DLL4 in tip cells. DDL4 in turn binds to and activates NOTCH1 on stalk cells, reducing VEGFR2 expression and subsequently increasing VEGFR1, thus constituting a negative feedback for the activity of VEGFA [7].

In the last several years, the development of targeted therapies has provided therapeutic options for patients with metastatic CRC in addition to improved clinical outcomes. The median overall survival (OS) has increased by > 12 months since the introduction of therapies using biological compounds and doublet/triplet chemotherapy regimens [8]. Bevacizumab (Avastin: Genentech, San Francisco, CA, USA), a monoclonal antibody, is a potent inhibitor of vascular endothelial growth factor (VEGF), and has been shown to reduce angiogenesis [9]. The efficacy and safety of bevacizumab have been demonstrated as both a first and second-line treatment [10–12]. In combination with chemotherapy, bevacizumab has been shown to improve the overall response rate (RR), median progression-free survival (PFS) and median OS [13]. The majority of previously untreated metastatic CRC patients are now treated with bevacizumab in combination with oxaliplatin and fluorouracil (FOLFOX) [14]. Furthermore, recent data have shown some clinical benefit in maintaining VEGF inhibition with bevacizumab beyond disease progression [15].

Although significant improvements in outcome rates have been reported with various anti-VEGF agents, a substantial number of patients do not obtain a pronounced benefit, which is most likely due to resistance mechanisms [16]. DLL4-induced Notch signaling, one of the mechanisms reported to mediate tumor resistance related to anti-VEGF therapy, activates multiple parallel pathways and induces the formation of large distorted vessels [7]. The Notch signaling pathway has emerged as an attractive target for angiogenesis-based cancer therapies [17]. However, despite the increasing role of various antiangiogenic drugs in personalized metastatic CRC care, no biomarkers have been identified capable of predicting response to antiangiogenic therapy. Several markers have been tested in preclinical models but failed as predictors of response in human trials [18]. Thus, there is an urgent need for prognostic/predictive biomarkers for anti-VEGF therapies.

In this retrospective study, clinical features and outcomes in a cohort of 105 CRC patients who received bevacizumab-containing regimens were reviewed and presented. The expression of VEGF receptors, NOTCH1 receptor and its ligand DLL4 in CRC tissues as well as the evaluation of their relationships with clinicopathological characteristics and outcomes were determined retrospectively. Importantly, the patient population was relatively homogeneous due to consistent eligibility criteria, treatment guidelines, and evaluation parameters.

## Methods

### Patients and study design

**Fig. 1:**
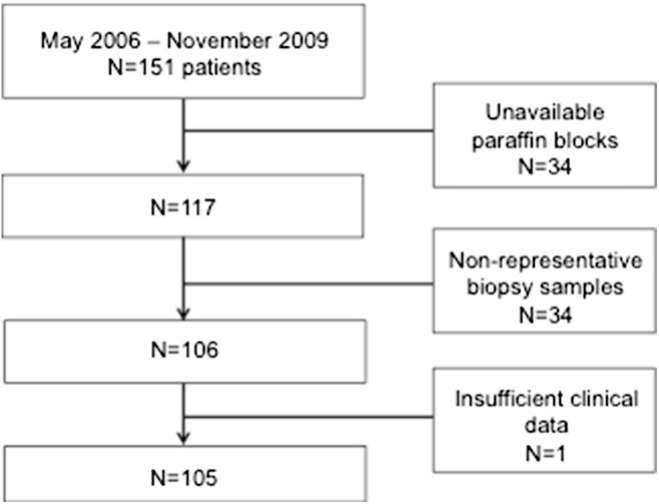
Flow diagram for patient selection

The medical data from CRC metastatic patients treated with a combination of bevacizumab and chemotherapy were retrospectively analyzed. All data from May 2006 to November 2009 were obtained from chemotherapy registries from A.C. Camargo Cancer Center - Fundação Antônio Prudente. A total of 151 patients were found. Paraffin blocks were available for 117 patients, but in 11 of them the data or material were not suitable for analysis, therefore, a total of 105 patients were eligible for further study (Fig. 1). The study was approved by the Ethics Committee of A.C. Camargo Cancer Center - Fundação Antônio Prudente (number 1134/08). The need of an informed consent was waived by Ethics Committee.

Demographic and clinical data were collected and included relevant medical history, disease stage, tumor pathology at the initial diagnosis, details of chemotherapy regimens used with bevacizumab, data concerning the primary surgery, and metastasectomy. Tumors were staged according to the 7th edition of the TNM classification of malignant tumors [19]. Data for the response criteria and survival outcomes were based on the chart review. Response criteria were evaluated according to the Response Evaluation Criteria in Solid Tumors version 1.1 (RECIST v1.1) [20]. Cases treated before the publication of RECIST v1.1 were classified accordingly based on data extracted from image reports. The PFS was defined as the time from the beginning of bevacizumab treatment until the first observation of disease progression. The OS was defined as the time from the beginning of bevacizumab treatment until the date of either the last contact alive or death from any cause. Hypertension was defined when blood pressure was either persistently elevated (>24 h) or repeated blood arterial pressure measurements were above 140 × 90 mmHg. The Common Terminology Criteria for Adverse Events (CTCAE) version 4.03 was used to grade hypertension [21]. Blood pressure values recorded by physicians and nurses during screening consultations, pre-chemotherapy, and prior to initiation of the antihypertensive treatment were analyzed. Archival pathological specimens were collected, and the expression of the angiogenesis-related proteins was first verified by immunohistochemistry and then quantified.

### Construction of tissue microarray blocks and immunohistochemistry analysis

Archival formalin-fixed, paraffin-embedded specimens were obtained from patients who received bevacizumab. The chosen samples were from primary tumor and/or metastatic tissue collected as close as possible to the start of bevacizumab treatment. The paraffin blocks underwent tissue microarray construction before immunostaining. In brief, a fresh section stained with hematoxylin and eosin was cut from each block. The representative tumor areas were carefully selected and marked. Using a tissue microarrayer (Beecher Instruments*,* Silver Spring, MD, USA), 1 mm cylindrical cores were removed from each donor paraffin block and transferred to premolded recipient paraffin blocks, in duplicates. Sections 5 μm in thickness were placed on glass slides. In the recipient block, cores were arrayed according to the defined x-y coordinate position. Normal placenta tissue cores were used as a position marker. Slides were then incubated with the primary antibodies according to the manufacturer’s protocol. The polyclonal antibodies used in this study were: PlGF (1:20, R&D systems, Minneapolis, MN, USA), VEGFR2 (1:50, Neomarkers, Freemont, CA, USA), VEGFR3 (1:400, LabVision, Freemont, CA, USA), and DLL4 (1:200, Abcam, Cambridge, UK). The monoclonal antibodies used were VEGFR1 (1:50, clone Y103, Abcam, Cambridge, UK) and NOTCH1 (1:50, Thermo Scientific, clone A6, Rockford, IL, USA). Antibody detection was performed using a streptavidin-biotin system (Biotinylated Link Universal, LSAB+, Carpinteria, CA, USA) for PlGF and a biotin-free polymeric visualization system (Novolink Max Polymer, Carpinteria, CA, USA) for all the other antibodies, according to the manufacturer’s protocol. Glass slides were digitalized using the Aperio Scan-Scope XT Slide Scanner (Aperio Technologies, Vista, CA, USA) at 20x magnification. All the tumoral areas in the tissue microarray (spots) were evaluated and scored independently by the pathologist (M.M.P) and the oncologist (T.F.P.J.), without previous knowledge of the clinicopathological outcomes of the patients. The evaluation of the immunostaining was as follows: VEGFR1 (membrane and cytoplasm), VEGFR2 (membrane and cytoplasm), VEGFR3 (membrane and cytoplasm), PlGF in the cytoplasm, and DLL4 and NOTCH1 in the membrane. A membrane staining algorithm (Membrane v1, Aperio, Vista, CA, USA) was used to determine the intensity and extent of cell membrane staining. Tumor cells with weak or partial membrane staining were scored 1+; tumor cells with moderate and complete membrane staining were considered 2+; tumor cells with intense and complete membrane staining were classified as 3+. For each TMA core, the percentage of cells with score 0, +1, +2, +3 was registered. A positive staining was considered for cells with scores 2+ and 3+, except for DLL4, where a score of 1+, 2+ and 3+ were considered positive. The percentage of cells with positive staining in each TMA core was summed up. The mean value per replicate was used for the statistical analysis. A sample was considered non-representative when there were <500 analyzed cells. For the quantification of stain in the cytoplasm, the Positive Pixel Count Algorithm (Aperio, Vista, CA, USA) was used to sum the strongly and moderately positive pixels in each core. The analyses included the classification of staining as strongly, moderately and weakly positive, the number of negative cells, the analyzed area, and the ratio of the number of positive/total number of cells. The mean value per replicate was used for statistical analyses. A sample was considered non-representative when there was an area <0.08 μm^2^ (10 % of the total core area).

### Statistical analysis

Descriptive statistics was used for the analysis of demographic and clinical characteristics. Frequencies and percentages were used for nominal/ordinal variables, while median and range were used for continuous variables. The response rates associated with demographic and clinical data were analyzed with the Chi-square and Fisher’s exact tests. Logistic regression was used to test the independent effect of some variables on the objective response rate; all variables with *p* ≤ 0.05 were included in association tests. Correlations among angiogenesis-related proteins employed a Pearson’s correlation test. The survival curves were estimated using the Kaplan-Meier method and were compared using the log-rank test. Patients who were lost to follow-up were censored at the last contact date. Ordinal or nominal variables were dichotomized by grouping. To analyze the association of OS and PFS with angiogenesis-related protein expression, patients were subdivided into high or low-expression groups based on the median cut-off values. The Cox multivariate regression model was used to test for independent significance of clinical and pathological parameters on the OS.

All analyses were performed either with the R statistical software, version 2.3.0 (www.r-project.org) or SPPS v.17 statistical package (Chicago, IL, USA).

## Results

### Patient and tumor characteristics

Median age at diagnosis was 56 years (range, 28–80 years); 58 % of patients were male, and 51 % of patients presented one or more comorbidities. The most common CRC localization was descending/sigmoid colon. Although all tumors were adenocarcinomas, 9.6 % presented mucinous histology (mucinous and mucin-secreting) and 82.6 % were moderately differentiated. By the time of diagnosis, 72.8 % of patients had stage IV (synchronous metastases), the remaining patients developed metachronous metastases (27.2 %), the liver being the most common site of metastasis. Ninety-five percent of patients underwent resection of the primary tumor, and 58 % underwent metastasis resection at some point. Tumor characteristics are presented in Table 1.

**Table 1:** Tumor characteristics

Characteristics	Frequency
Localization (*n* = 104)	
Descending/sigmoid colon	57.7 %
Medium and lower rectum	22.1 %
Ascending colon	17.3 %
Transverse colon	2.9 %
Histology (*n* = 104)	
Adenocarcinoma NOS	50.0 %
Tubular adenocarcinoma	39.4 %
Mucin-secreting adenocarcinoma	4.8 %
Mucinous adenocarcinoma	3.8 %
Signet ring cell carcinoma	1.0 %
Cribriform adenocarcinoma	1.0 %
Histologic differentiation (*n* = 92)	
Well (1)	6.5 %
Moderate (2)	82.6 %
Poor (3)	10.9 %
Blood vessel invasion (*n* = 81)	
Yes	16.0 %
No	84 %
Lymphatic invasion (*n* = 80)	
Yes	36.3 %
No	63.7 %
Perineural invasion (*n* = 78)	
Yes	30.8 %
No	60.2 %
Depth of tumor invasion (*n* = 98)	
T1	2.0 %
T2	6.1 %
T3	75.5 %
T4	15.3 %
Tx	1.0 %
Lymph node metastasis (*n* = 98)	
N0	30.6 %
N1	32.7 %
N2	35.7 %
Nx	1.0 %
Metastasis (M1) at diagnosis (*n* =104)	
Yes	72.8 %
No	27.2 %
TNM staging at diagnosis (*n* =103)	
I	1.0 %
II	11.7 %
III	14.6 %
IV	72.8 %
Metastatic sites (*n* = 105)	
Liver	65.7 %
Lymph nodes	25.7 %
Lung	33.3 %
Peritoneum	17.1 %
Skin/subcutaneous	1.0 %
Locoregional recidive	11.5 %
Metastasis resection (*n* = 105)	
Yes	58.1 %
No	41.9 %

n: number of patients with available data in each category. Tumors were staged according to the 7th Edition of the TNM Classification of Malignant Tumors. NOS: not otherwise specified

Although most of the patients had an advanced stage of the disease at diagnosis, 96.9 % presented an ECOG (Eastern Cooperative Oncology Group) performance status of either 0 or 1. Bevacizumab was administered as the first, second, third, and even the fourth line of therapy, with the majority of cases (70 %) using it as the first-line. Irinotecan plus 5-fluorouracil and leucovorin was the most common chemotherapy drug regimen associated with bevacizumab (53.3 %). Hypertension is a common side effect of bevacizumab therapy. In our cohort, 25.8 % of patients experienced chemotherapy-related hypertension grade 2 or 3.

### Clinical outcomes

**Fig. 2:**
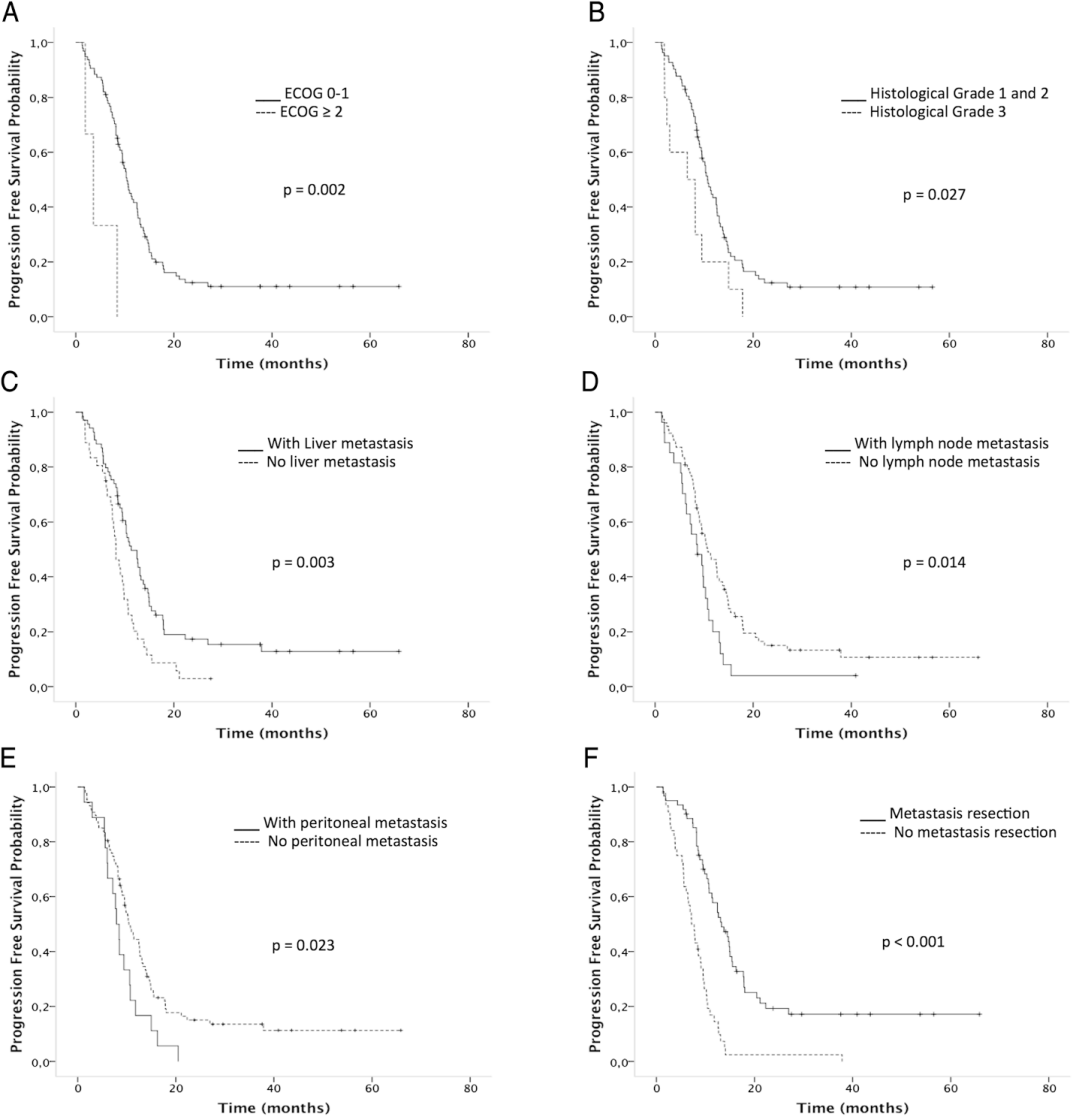
The progression-free survival curves according to: ECOG (1 = ECOG 0, 1 vs. 2 = ECOG 2, 3), *p* = 0.002 (**a**); histogical grade (well differentiated = grade 1, moderately differentiated = grade 2 vs. poorly differentiated = grade 3), *p* = 0.0027 (**b**); with liver metastasis (yes vs. no), *p* = 0.003 (**c**); lymph node metastasis (yes vs. no), *p* = 0.014 (**d**); peritoneal metastasis (yes vs. no), *p* = 0.023 (**e**), and patients who underwent metastasis resection (yes vs. no), *p* < 0.001 (**f**). The survival curves were calculated using the Kaplan-Meier method, and the log-rank test was used for comparison. ECOG: Eastern Cooperative Oncology Group performance status score

In the total cohort of 105 patients, the median PFS was 10.2 months. There was a significant association between PFS and ECOG 0 or 1 (*p* = 0.002), and PFS and histological differentiation grades 1+ and 2+ (*p* = 0.027), shown in Fig. 2a and b. Concerning the presence of metastasis, a longer PFS was significantly associated with the presence of liver metastasis (*p* = 0.003, Fig. 2c), absence of lymph node metastasis (*p* = 0.014, Fig. 2d), absence of peritoneal metastasis (*p* = 0.023, Fig. 2e), and with patients undergoing metastasis resection (*p* < 0.001, Fig. 2f).

**Fig. 3:**
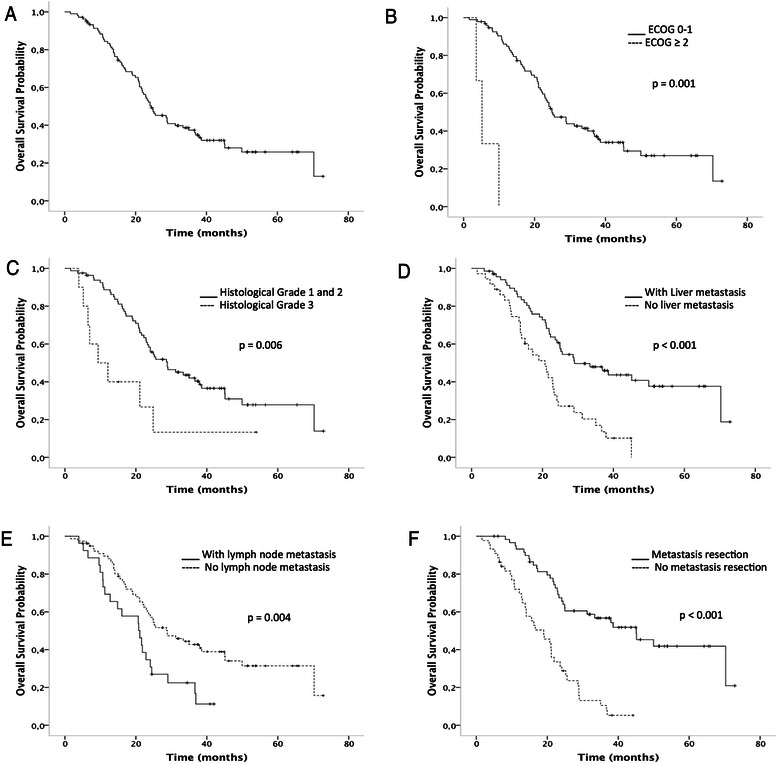
The overall survival curves from the start of bevacizumab treatment. Overall survival curve from the total cohort (*n* = 105 patients), median of 24.4 months (**a**). Overall survival curves according to: ECOG (1 = ECOG 0, 1 vs. 2 = ECOG 2, 3), *p* = 0.001 (**b**); histogical grade (well differentiated = grade 1, moderately differentiated = grade 2 vs. poorly differentiated = grade 3), *p* = 0.006 (**c**); liver metastasis (yes vs. no), *p* < 0.001 (**d**); lymph node metastasis (yes vs. no), *p* = 0.004 (**e**), and patients who underwent metastasis resection (yes vs. no), *p* < 0.001 (**f**). The survival curves were calculated using the Kaplan-Meier method, and the log-rank test was used for comparison. ECOG: Eastern Cooperative Oncology Group performance status score

The median OS of the total cohort was 24.4 months (Fig. 3a). There was a significant association between better OS and ECOG 0 or 1 (*p* = 0.01, Fig. 3b). Improved OS was also associated with histological differentiation grades 1+ or 2+ (*p* = 0.006, Fig. 3c), presence of liver metastasis (*p* < 0.001, Fig. 3d), absence of lymph node metastasis (*p* = 0.004, Fig. 3e), and with patients undergoing metastasis resection (*p* < 0.0001, Fig. 3f). Patients who underwent metastasis resection at some point in the course of their disease had better OS (5*-*year survival rate of 40 %) as well as better PFS, with a10-year survival rate of 18 %.

Response rates were analyzed in 95 patients since nine patients did not present detectable disease prior to bevacizumab treatment and one patient had no data regarding response to treatment. Association with clinical variables is shown in Table 2. Histological differentiation grades 1+ and 2+, non-mucinous histology, liver metastasis and first-line therapy were significantly associated with better objective response rates. In a multivariate logistic regression model for objective response the included variables were line of bevacizumab treatment (first vs. others), combined histology (mucin-secreting vs. non-mucin-secreting), histological differentiation grade (1, 2 vs. 3) and the presence of liver metastasis (yes vs. no). Bevacizumab, used in the first-line of treatment, and the presence of liver metastasis were independently associated with objective response rate (Table 3).

**Table 2:** Association between clinical variables and objective response by RECIST

Variable		Total n	CR + PR *n* (%)	SD + PD *n* (%)	*p value*
Grade	1 + 2	74	42 (57)	32 (43)	0.01
	3	9	1 (11)	8 (89)	
Mucinous histology	no	84	46 (55)	38 (45)	0.048
	yes	10	2 (20)	8 (80)	
Liver metastasis	no	31	8 (26)	23 (74)	0.001
	yes	64	40 (62)	24 (38)	
Peritoneal metastasis	no	79	43 (54)	36 (46)	0.09
	yes	16	5 (31)	11 (69)	
Chemotherapy	FOLFIRI	52	25 (48)	27 (52)	0.43
	FOLFOX	39	22 (56)	17 (44)	
Hypertension	no	61	29 (47)	32 (53)	0.41
	yes	30	17 (57)	13 (43)	
Chemo + bevacizumab as first-line therapy^a^	no	28	9 (36)	19 (64)	0.02
	yes	67	40 (60)	27 (40)	

RECIST: Response Evaluation Criteria in Solid Tumors; FOLFIRI: infusional 5-fluoro-uracyl plus leucovorin plus irinotecan; FOLFOX: infusional 5-fluoro-uracyl plus leucovorin plus oxaliplatin; CR: Complete Response; PR: Partial Response; SD: Stable Disease; PD: Progressive Disease (PD). Data are number of patients and numbers in parentheses are percentages. ^a^Chemo: chemotherapy

**Table 3:** Multivariate logistic regression analysis showing the variables independently associated with objective response rate (*n* = 83 patients)

Covariates	Odds ratio	CI 95 %	*p value*
Chemo + bevacizumab^a^			
First-line vs. others	3.65	1.03–12.87	0.032
Liver metastasis			
yes vs. no	4.12	1.18–14.29	0.027
Histological grade			
G1, 2 vs. G3	9.78	0.97–98.98	0.059
Histology			
Mucin-secreting vs. non-mucin-secreting	0.47	0.03 – 6.18	0.56

CI: Confidence interval. ^a^Chemo: chemotherapy

We also evaluated ORR, PFS and OS from patients that received bevacizumab as a first-line (*N* = 73) and second-line treatment (*N* = 29) separately. Demographics and clinical characteristics of patients treated with bevacizumab as a first-line therapy are shown in (Additional file 1: Table S1). ORR, median PFS and median OS was 59.7 %, 10.6 months and 28.9 months in first-line and 36.0 %, 8.2 months and 19.9 months in second-line, respectively (Additional file 1: Table S2).

Angiogenesis-related proteins were investigated by immunohistochemistry in patients with the disease detected prior to bevacizumab treatment (Additional file 2: Figure S1). The proteins expressed most abundantly were VEGFR1 and NOTCH1. The expression values for VEGFRs, PlGF, DLL4 and NOTCH1 are presented in Table 4. All of the VEGF receptors 1, 2 and 3 had significant positive correlations among each other for both membrane and cytoplasmic expression. All other significant correlations involving PlGF, DLL4 and NOTCH1 are presented in Table 5.

**Table 4:** Expression of angiogenesis-related proteins in the total cohort

Angiogenesis-related protein	*n*	% of positive cells/core (median)	Range (%)
VEGFR1			
Membrane	96	64.5	1 – 76
Cytoplasm	96	60.4	0 - 86.7
VEGFR2			
Membrane	96	9.5	0 – 64
Cytoplasm	92	22	0.35 – 72.8
VEGFR3			
Membrane	96	17	0 – 67
Cytoplasm	94	26.3	2.06 – 80.1
PlGF	93	38.9	0.44 – 83.1
DLL4	92	27	1 – 99
NOTCH1	96	69	6 – 79

**Table 5:** Correlations between angiogenesis-related proteins

Angiogenesis-related proteins	Correlation coefficient	*p*
PlGF and membrane VEGFR1	0.50	<0.0001
PlGF and cytoplasm VEGFR1	0.58	<0.0001
PlGF and cytoplasm VEGFR2	0.23	<0.0001
DLL4 and membrane VEGFR1	0.28	0.0243
DLL4 and cytoplasm VEGFR1	0.28	0.0056
DLL4 and PLGF	0.24	0.0172
DLL4 and NOTCH1	0.25	0.0161
NOTCH1 and membrane VEGFR1	0.56	<0.0001
NOTCH1 and cytoplasm VEGFR1	0.34	<0.0001
NOTCH1 and PlGF	0.27	0.0072

The clinical characteristics significantly associated with angiogenesis-related proteins are described in Table 6. DLL4 did not show any significant association with clinical characteristics. There was no significant association between objective response rate and the angiogenesis-related proteins investigated.

**Table 6:** Association between expression of angiogenesis-related proteins and clinical characteristics

	Characteristic	*p*
VEGFR1		
Membrane	Lung metastasis	0.002
Cytoplasm	Stage I and II	0.03
VEGFR2		
Cytoplasm	Stage I and II	0.03
VEGFR3		
Membrane	Lung metastasis	0.001
	Liver metastasis	0.03
Cytoplasm	Lung metastasis	0.003
PlGF	Stages I and II	0.04
NOTCH1	Metastasis to regional lymph nodes (N1 or N2)	0.04

**Fig. 4:**
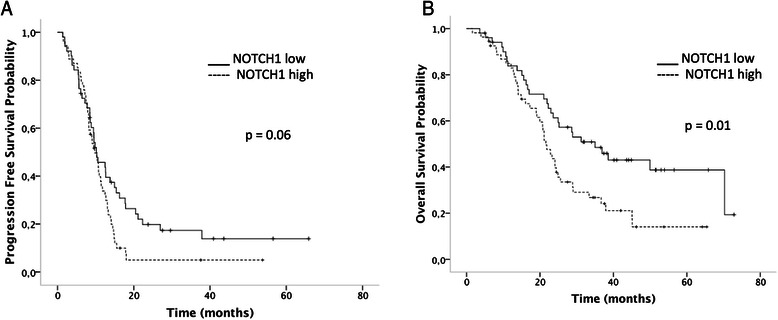
The progression-free survival curve for NOTCH1 expression, *p* = 0.06 (**a**), and the overall survival for NOTCH1, *p* = 0.01 (**b**). Low expression: < median expression value vs. high expression: ≥ median expression value. The survival curves were calculated using the Kaplan-Meier method, and the log-rank test was used for comparison

There was a trend toward an association between better PFS with lower NOTCH1 expression (*p* = 0.06, Fig. 4a). Better OS was significantly associated with lower NOTCH1 expression (*p* = 0.01, Fig. 4b). To determine the variables independently associated with OS, ECOG (0, 1 vs. 2, 3), metastasis resection (yes vs. no), lymph node metastasis (yes vs. no), peritoneal metastasis (yes vs. no), liver metastasis (yes vs. no), histological grade (1, 2 vs. 3), and NOTCH1 expression (lower vs. higher) were included in a multivariate analysis shown in Table 7.

**Table 7:** Multivariate regression analysis for overall survival (OS). Eighty-eight patients with complete data were included in this analysis

Covariates	HR	CI 95 %	*p*
Liver metastasis (yes vs. no)	1.93	1.09 – 3.44	0.024
Histological grade (1 + 2 vs. 3)	2.79	1.16 – 6.68	0.021
ECOG (0 + 1 vs. 2 + 3)	20.50	3.08 – 136.11	0.002
NOTCH1	2.01	1.07 – 3.77	0.029

HR: Hazard ratio; ECOG: Eastern Cooperative Oncology Group performance status score; CI: Confidence interval

## Discussion

This retrospective analysis reports on the efficacy of bevacizumab administered across multiple lines of treatment in a large cohort of patients from a reference center in Brazil. We report the expression profile of angiogenesis-related proteins and their association with clinical characteristics and outcomes.

Several phase III clinical trials have confirmed the efficacy of bevacizumab in combination with FOLFOX or FOLFIRI in the first-line treatment for metastatic CRC [10, 22]. In 100 patients receiving, as the first-line therapy, bevacizumab with irinotecan, 5-fluorouracil and leucovorin, a median PFS of 8.8 months was reported compared to 6.8 months in the group without bevacizumab [10]. Reported PFS in this setting varied from 7 to 11 months [11, 22, 23]. In our cohort of 105 patients, 70.0 % received bevacizumab in the first-line treatment with the median PFS of 10.6 months. Although this cohort included a high proportion of patients with advanced disease, our results are in line with reported data confirming the clinical value of bevacizumab. As to be expected, a significantly better PFS was associated with ECOG 0 or 1 and histological differentiation grades 1+ and 2+. Additionally, a better PFS was significantly associated with the absence of extrahepatic metastasis and the presence of liver metastasis, an outcome probably related to the high rate of metastasis resection (58.1 %). Our data showed a median OS of 24.4 months, in line with the literature and associated with similar factors that were also related to a better PFS, such as liver metastasis and metastasis resection [12, 22, 24, 25]. Concerning objective responses, better response rates were seen in histological differentiation grades 1+ and 2+, non-mucinous histology, liver metastasis, and bevacizumab as the first-line therapy. However, only the last two variables were independently associated. Importantly, the objective response rate was 59 % in patients who used bevacizumab as the first-line therapy (67 patients in this analysis).

We analyzed the occurrence of hypertension, the most common side effect of bevacizumab. Hypertension appears to be dose-dependent, and it is currently under an investigation as a biomarker for VEGF inhibition efficacy [26, 27]. Bevacizumab-induced hypertension has been reported to be highly associated with improvements in PFS, OS, and the overall RR [27]. In our study, hypertension grades 2 and 3 were present in 25.8 % of patients. Grade 3 hypertension was present in 13.9 %, in line with previously reported data [28]. However, we did not identify a significant association between the presence of hypertension and objective response rate. No significant association was seen between outcomes or expression of angiogenesis-related proteins and hypertension.

Although outcome results may vary, the use of bevacizumab therapy has been well established [25, 29]. However, the heterogeneity of results shown in several clinical trials demonstrates an urgent need for biomarkers predicting bevacizumab treatment outcome and toxicity. Different biomarkers such as mutations of *BRAF* and *KRAS* [30], microvessel density and VEGF levels [31] have been studied, but no predictive factors have been identified. Biomarkers would allow for the selection of patients most prone to respond to antiangiogenic therapy. In this cohort, the most expressed angiogenesis-related proteins were VEGFR1 and NOTCH1 with a median value of ~ 65 % positive cells.

PlGF is a VEGF homolog and a ligand for VEGFR1 that acts by amplifying the responsiveness of VEGFR1 to VEGF during pathological angiogenesis [32]. NOTCH1 signaling activation has been shown to induce VEGFR1 expression [33] and enhance its responsiveness to PlGF [34]. In line with this, we found significant positive correlations between the expression of VEGFR1 (membrane and cytoplasm) and PlGF, as well as between VEGFR1 (membrane and cytoplasm) and NOTCH1, and NOTCH1 and PlGF. However, although there is a negative NOTCH1 signaling feedback to VEGFR2, resulting in decreased endothelial cell proliferation in response to VEGF [35], there was no significant correlation between the expression of NOTCH1 or DLL4 and VEGFR2. While bevacizumab treatment has been associated with increased levels of PlGF [36], its value as a biomarker remains largely unknown. Instead of contributing to tumor escape and resistance, as has been suggested, increased PlGF expression might only be a host response elicited by angiogenic therapies [37] since it has been associated with better outcomes in glioblastoma [38].

We also analyzed the association of angiogenesis-related protein expressions with clinical and pathological characteristics. In tumors or plasma, VEGFR1 is a strong biomarker candidate to predict response to bevacizumab [39]. The median expression of VEGFR1 was twice that of VEGFR2 in our cohort, and higher expression of membrane VEGFR1 was associated with lung metastasis. In the MAX III trial (testing the combination of capecitabine, bevacizumab and mitomycin), the authors observed an association between low VEGFR1 expression by immunohistochemistry on tumors and improved OS [39].

VEGFR3 is a major inducer of lymphangiogenic signaling, but its expression has been associated with the presence of hematogenous metastasis [40] and poor OS [41]. In our cohort, while higher expression of membrane and cytoplasmic VEGFR3 was associated with lung metastasis, lower expression was associated with liver metastasis. YILDIZ et al. (2010) evaluated the expression of VEGFR3 by immunohistochemistry in tumor samples from metastatic colorectal cancer patients treated with chemotherapy and bevacizumab and observed positive expression in 20 % of cases. The authors did not identify any association with efficacy outcomes [42].

Increased NOTCH1 expression was associated with lymph node metastasis, which is in line with previously reported data [43, 44].

PFS was not significantly associated with any of the angiogenesis-related proteins. We saw a trend toward longer PFS in patients whose tumors bore lower expression of NOTCH1, most likely because NOTCH1 expression might be involved in tumor resistance to bevacizumab. Angiogenesis induced by DLL4-NOTCH1 signaling generates large vessels that increase tumor blood supply and diminish sensitivity to bevacizumab [16]. Besides, lower expression of NOTCH1 was significantly associated with better OS. NOTCH1 activation induces VEGFR3 [5], and it has already been associated with poor survival [43]. In multivariate analysis, the only factors that were independently associated with PFS were histological grade and metastasis resection. For OS, NOTCH1 remained an independent variable, confirming the reported association between overactivated NOTCH signaling and poor survival in CRC [43]. CHU et al. described that, among 1003 tumor samples from early stage resected colorectal cancer, 52.1 % of cases were positive for NOTCH1 and 33.4 % for NOTCH2 by immunohistochemistry. Patients bearing NOTCH1 positive tumors had worse OS (38 months vs 66 months for NOTCH1 negative cases). The opposite results were observed in association with NOTCH2 expression, where low NOTCH2 was associated with worse OS [45]. Similarly, we also found that NOTCH1 hyperexpression is associated with reduced OS, however differently from the studies by CHU et al. (2011) [46, 47], our cohort is composed solely of metastatic colorectal cancer patients treated with chemotherapy plus bevacizumab. Although, we can not draw conclusions on the predictive capacity of NOTCH1, we believe it is definitely prognostic, and, to our knowledge, this is the first published report showing this association in this population.

In summary, the results of this retrospective study are similar to the outcomes observed in large clinical trials confirming the effectiveness of bevacizumab in common daily practice. The expression profile of angiogenesis-related proteins and their association with clinicopathological characteristics and outcomes were described, building on the current understanding of the role of these proteins in the setting of chemotherapy in addition to bevacizumab therapy. Our results suggest that NOTCH1 might serve as a prognostic marker for colorectal cancer in the metastatic setting.

## Conclusion

We evaluated the expression of proteins involved with angiogenesis in colorectal tumor samples from patients receiving bevacizumab in conjunction with chemotherapy. High expression levels of VEGFR1 and VEGFR3 were associated with a higher rate of lung metastasis. VEGFR3 expression was also associated with liver metastasis. NOTCH1 expression was associated with an increased risk of lymph node metastasis and a worse overall survival.

## Additional files

Additional file 1: Table S1. Demographic and clinical characteristics of patients receiving chemotherapy plus bevacizumab as first-line treatment (*N* = 73). **Table S2.** Data regarding efficacy outcomes according to line of treatment. Table depicts overall response rate (ORR), PFS (progression free survival) and overall survival (OS) of patients treated with chemotherapy plus bevacizumab in first-line in comparison to second-line. (DOCX 81 kb)
Additional file 2: Figure S1. Microphotographs displaying the pattern of immunohistochemical protein expression observed for each studied protein. For each marker, a positive and a negative case is shown. **A**) and **B**) VEGFR1; **C**) and **D**) VEGFR2; **E**) and **F**) VEGFR3; **G**) and **H**) PLGF; **I**) and **K**) NOTCH1, and **L**) and **M**) DLL4. (PPTX 4454 kb)

## Abbreviations

BRAF: v-RAF murine sarcoma viral oncogene homolog B
CI: Confidence interval
CRC: Colorectal cancer
DLL4: Delta-like ligand
ECOG: Eastern Clinical Oncology Group
FOLFIRI: Folinic acid + 5-fluorouracyl + irinotecan
FOLFOX: Folinic acid + 5-fluorouracyl + oxaliplatin
HR: Hazard ratio
KRAS: Kirsten rat sarcoma viral oncogene homolog
NOS: Not otherwise specified
NOTCH1: Neurogenic locus notch homolog protein 1
OS: Overall survival
PFS: Progression free survival
PlGF: Placental growth factor
RECIST: Response evaluation criteria in solid tumors
TMA: Tissue microarray
TNM: Tumor, node and metastasis
VEGF: Vascular endothelial growth factor
VEGFR: Vascular endothelial growth factor receptor
