# Supplementary material for: Angiogenesis-related protein expression in bevacizumab-treated metastatic colorectal cancer: NOTCH1 detrimental to overall survival
Source: BMC Cancer. 2015 Sep 22;15:643. doi: 10.1186/s12885-015-1648-4 (PMC4579833; doi:10.1186/s12885-015-1648-4)
Supplement: Additional file 1: Table S1. — Demographic and clinical characteristics of patients receiving chemotherapy plus bevacizumab as first-line treatment (N = 73). Table S2. Data regarding efficacy outcomes according to line of treatment. Table depicts overall response rate (ORR), PFS (progression free survival) and overall survival (OS) of patients treated with chemotherapy plus bevacizumab in first-line in comparison to second-line. (DOCX 81 kb) [file 12885_2015_1648_MOESM1_ESM.docx]

**SUPPLEMENTARY ONLINE DATA**

**Table S1 -** Demographic and clinical characteristics of patients receiving chemotherapy plus bevacizumab as first-line treatment (N=73).

| **Variable (N=number of patients with available data)** |  |
| --- | --- |
| **Age (years)**  Median | 57 (28-80) |
| **Gender (N= 73)**  Male  Female | 56%  44% |
| **Tumor location (N= 73)**  Left colon  Descending / sigmoid / upper rectum  Medium and lower rectum  Right colon  Ascending  Transverse | 82%  59%  23%  18%  15%  3% |
| **Histology (N= 72)**  Adenocarcinoma NOS  Tubular adenocarcinoma  Mucin-secreting adenocarcinoma  Mucinous adenocarcinoma  Signet ring cell adenocarcinoma  Cribriform adenocarcinoma | 47%  47%  3%  3%  0%  0% |
| **Histological grade (N= 66)**  1  2  3 | 6%  86%  8% |
| **Stage at diagnosis (N=72)**  I  II  III  IV | 0%  15%  13%  72% |
| **Sites of metastatic disease (N= 73)**  Liver  Lung  Lymph node  Peritonium  Locorregional relapse | 68%  27%  22%  19%  10% |
| **Resection of the primary tumor (N= 73)**  Yes  No | 96%  4% |
| **Neoadjuvant radiotherapy (N= 73)**  Yes  No | 20%  80 |
| **Neoadjuvant chemotherapy (N= 73)**  Yes  No | 19%  81% |
| **Adjuvant chemotherapy* (N= 73)**  Yes  No | 23%  85% |
| **Adjuvant chemotherapy protocol (N=17)**  FOLFOX / FLOX  5-FU / Capecitabine | 71%  24% |
| **Resection of metastatic disease (N= 73)**  Yes  No | 57%  43% |
| **ECOG^#^ (N= 70)**  0  1  2  3 | 76%  23%  0%  1% |
| **Measurable disease at bevacizumab start time (N= 73)**  Yes  No | 92%  8% |
| **Chemotherapy protocol given with bevacizumab (N= 73)**  FOLFOX  FOLFIRI  IROX | 56%  41%  3% |
| **Number of chemotherapy cycles**  Median | 12 (2-21) |
| **Number of bevacizumab cycles**  Median | 11 (2-21) |
| **Bevacizumab schedule (N=73)**  5mg/Kg biweekly  7.5mg/Kg monthly | 99%  1% |
| **Arterial hypertension (N= 70)**  0  1  2  3  4 | 69%  4%  13%  14%  0% |
| **KRAS exon 2 mutation (N=58)** | 35% |

NOS: not otherwise specified. * Seventeen patients received adjuvant chemotherapy at the time of primary tumor diagnosis. ^#^ ECOG was evaluated at the time of bevacizumab initiation.

**Table S2.** Data regarding efficacy outcomes according to line of treatment. Table depicts overall response rate (ORR), PFS (progression free survival) and overall survival (OS) of patients treated with chemotherapy plus bevacizumab in first-line in comparison to second-line.

| **Outcomes** | **First-line** | **Second-line** |
| --- | --- | --- |
| **ORR** | 59.7% | 36.0% |
| **PFS** | 10.6 months | 8.2 months |
| **OS** | 28.9 months | 19.9 months |
