# Supplementary figures and images for: Angiogenesis-related protein expression in bevacizumab-treated metastatic colorectal cancer: NOTCH1 detrimental to overall survival
Source: BMC Cancer. 2015 Sep 22;15:643. doi: 10.1186/s12885-015-1648-4 (PMC4579833; doi:10.1186/s12885-015-1648-4)

## Slide 1
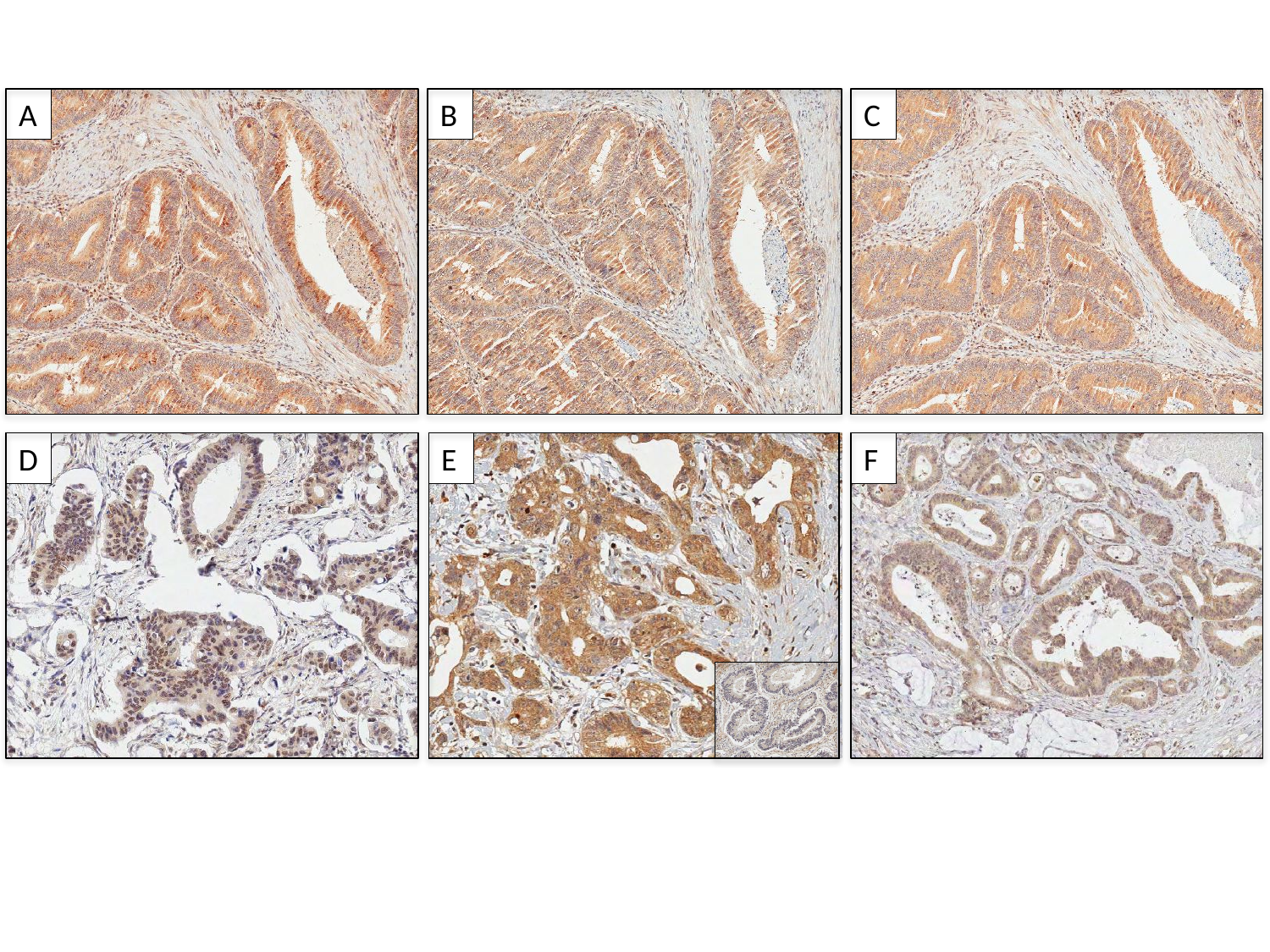

A
B
C
D
E
F

Supplement: Additional file 2: Figure S1. — Microphotographs displaying the pattern of immunohistochemical protein expression observed for each studied protein. For each marker, a positive and a negative case is shown. A) and B) VEGFR1; C) and D) VEGFR2; E) and F) VEGFR3; G) and H) PLGF; I) and K) NOTCH1, and L) and M) DLL4. (PPTX 4454 kb) [file 12885_2015_1648_MOESM2_ESM.pptx]
